# Single-Molecule Detection of DNA in a Nanochannel by High-Field Strength-Assisted Electrical Impedance Spectroscopy

**DOI:** 10.3390/mi10030189

**Published:** 2019-03-15

**Authors:** Porpin Pungetmongkol, Takatoki Yamamoto

**Affiliations:** 1 International School of Engineering, Faculty of Engineering, Chulalongkorn University, Bangkok 10330, Thailand; porpin.p@chula.ac.th; 2 Department of Mechanical Engineering, Tokyo Institute of Technology, Tokyo 152-8550, Japan

**Keywords:** single molecule, nanochannel, nanogap, electrical impedance spectroscopy, dielectrophoresis, electrophoresis, DNA

## Abstract

Many researchers have fabricated micro and nanofluidic devices incorporating optical, chemical, and electrical detection systems with the aim of achieving on-chip analysis of macromolecules. The present study demonstrates a label-free detection of DNA using a nanofluidic device based on impedance measurements that is both sensitive and simple to operate. Using this device, the electrophoresis and dielectrophoresis effect on DNA conformation and the length dependence were examined. A low alternating voltage was applied to the nanogap electrodes to generate a high intensity field (>0.5 MV/m) under non-faradaic conditions. In addition, a 100 nm thick gold electrode was completely embedded in the substrate to allow direct measurements of a solution containing the sample passing through the gap, without any surface modification required. The high intensity field in this device produced a dielectrophoretic force that stretched the DNA molecule across the electrode gap at a specific frequency, based on back and forth movements between the electrodes with the DNA in a random coil conformation. The characteristics of 100 bp, 500 bp, 1 kbp, 5 kbp, 10 kbp, and 48 kbp λ DNA associated with various conformations were quantitatively analyzed with high resolution (on the femtomolar level). The sensitivity of this system was found to be more than about 10 orders of magnitude higher than that obtained from conventional linear alternating current (AC) impedance for the analysis of bio-polymers. This new high-sensitivity process is expected to be advantageous with regard to the study of complex macromolecules and nanoparticles.

## 1. Introduction

Recent advances in nanotechnology have led to rapid growth in nanofluidics research based on fluid manipulations [1,2,3,4], reactions [5,6], analyses [7,8,9], and other processes in which the unique characteristics of nanometer-scale spaces and interfaces are utilized [7,10]. At present, unidimensional nanochannels are of special interest since they represent the only practical approach to the continuous processing of miniscule fluid samples, which may be necessary for the preparation, detection, separation, and reaction of samples in a single flow stream. Thus, nanochannels are expected to play an extremely important role as a platform for continuous single molecule and single nanoparticle processing. 

The realization of fully miniaturized continuous processing of single molecules or nanoparticles will allow the development of novel methods and applications that take advantage of the exceptional sensitivity and accuracy possible at this level of focus. The combination of this absolute sensitivity with continuous, portable instrumentation has allowed the development of various types of analytical chips, capable of monitoring different environments for viruses [11,12], bacteria [13] and particulate materials [14]. In addition, modules with applications to health care are expected to allow the analysis of sweat, saliva, tears, and other biological fluids, and breath analyzers for the detection of diabetes, various cancers, and additional diseases via the detection of specific molecules and aerosolized viruses in exhaled air have been demonstrated [15,16,17,18,19]. 

In anticipation of the emergence of these innovative applications in the near future, it is beneficial to consider the types of electrical measurements that are best suited to such electronic devices and that allow overall system miniaturization. In particular, recent progress in electrical detection systems now allows the direct measurement of molecules without complex modifications of either the sample or the measurement electrode surfaces. As an example, the single molecule detection of DNA in a nanochannel with embedded nano-gap electrodes was achieved by direct current measurements [20], while alternating current measurements based on electrical impedance have also allowed the detection of DNA in nano-gap electrodes [21,22]. Several types of virus particles have also been identified by electrical impedance spectroscopy using a nano-gap electrode [11]. However, there is an inherent problem associated with such systems in that sensitivity tends to decline as the analysis unit is miniaturized. This phenomenon represents an important bottleneck with regard to achieving practical applications of single-molecule and nanoparticle processes. Furthermore, conventional methods of performing electrical measurements of liquid samples suffer from several challenges resulting from unavoidable electrode reactions and decomposition processes. These include substantial changes in the measurement volume due to the decomposition of electrodes during the generation of faradaic currents, marked variations in the conductivity of liquid samples resulting from the release of ions from the electrode material, and the possible denaturation of the sample by reaction with these ions, as well as the generation of bubbles in the liquid sample. These and other issues that are negligible in macroscale measurement systems become significant in nanospaces. Therefore, it is necessary to find new electrical measurement methods that can both provide higher sensitivity and mitigate these deleterious effects.

In the present work, we developed a novel sensing method for single biomolecules based on electrical impedance spectroscopy (EIS) enhanced by taking advantage of the electric field in a nanospace. The advantages of this method include the facile generation of a very strong electric field in response to an applied voltage, which is sufficiently low so as to avoid electrode reactions, the induction of a high electric field by electrostatic forces to control changes in the motion, and conformation of the sample molecule as a means of amplifying the impedance signal, a short transit distance across the inter-electrode space that promotes good high frequency characteristics and reduces the faradaic current, and the minimal diffusion effects resulting from a unique measurement environment. All these effects allow measurements to be made with relative simplicity. As an example of the size effects in such a system, a 1 cm electrode gap requires 10 kV in order to obtain an electric field strength of 1 MV/m, whereas a 100 nm electrode gap requires only 0.1 V. Thus, a nanogap electrode enables measurements to be performed using a very high electric field while suppressing the electrode reactions described above. The present work was aimed at developing a novel high-electric field strength-enhanced EIS system. 

Herein, we describe the characteristics of EIS when utilizing the electrostatic forces generated by strong electric fields in a nanochannel. We also report the sensitivity obtained when using DNA molecules as samples, and demonstrate the single-molecule detection of DNA. This work represents a starting point for the development of single-molecule and nanoparticle processing based on electrical measurements, taking advantage of nanofluidics.

## 2. Materials and Methods

### 2.1. Measurement Device

**Figure 1:**
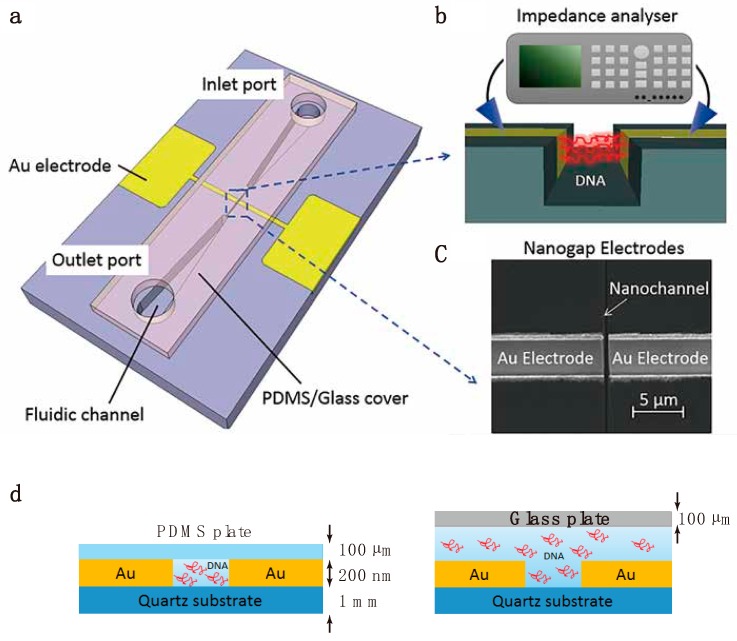
Measurement device and setup. (**a**) Schematic of the nanofluidic measurement device having a nanochannel located between two nanogap measurement electrodes. The electrodes are fully embedded in the quartz substrate to create a uniform electric field in the measurement area and to better seal the cover plate. Two types of cover plates, glass or polydimethylsiloxane (PDMS) (both 100 µm thick), were used to seal the nanochannel. (**b**) Schematic of the fluidic operation and electrical configuration of the measurement device. (**c**) Magnified scanning ion microscopy image of the measurement area. (**d**) The two cover plates used to assemble the device. The PDMS cover confined the DNA within the nanochannel to allow for quantitative electrical impedance measurements, whereas the glass cover was used to observe the dynamic behavior of DNA in the nanochannel.

Schematic illustrations of the measurement device and the experimental setup are presented in Figure 1. The device consists of an electrode chip incorporating a nanochannel sandwiched between two nanogap measurement electrodes, together with a cover plate to seal the electrode chip (Figure 1a). The sample molecules migrate through the nanochannel and are measured in the zone between the nanogap electrodes as they move through this region (Figure 1b). The design of this unit is similar to that of our previously fabricated nanofluidic device [23]. The fluidic channels are covered with a plate composed of a polydimethylsiloxane (PDMS) silicone elastomer capable of hermetically sealing the nanochannel, so as to confine the DNA sample molecules within the nanochannel, and this plate was used when measuring the electrical impedance. However, this PDMS cover plate could not be employed when observing the dynamic behavior of the DNA, since the swelling of the PDMS induced by the immersion oil required for the high magnification objective lens made it difficult to obtain clear images during fluorescent microscopy. To observe the dynamic behavior of DNA in the nanochannel, we therefore employed a glass cover plate (Figure 1d). Unfortunately, this plate did not perfectly seal the nanochannel but rather floated above the electrode chip. Thus, the DNA was not confined to the nanochannel, and so the measured electrical impedance was less precise compared to the values obtained with the PDMS cover plate. 

The nanochannel, with measurement electrodes at its center, was connected to the inlet and outlet ports through microchannels. The sample solution was injected into the inlet port and was then supplied to the measurement area either by capillary action or by the application of a vacuum to the outlet port to control the sample rate of motion. The measurements were conducted under stopped flow conditions to maintain a uniform concentration.

### 2.2. Materials

DNA samples of various lengths were prepared by separation from commercially-available DNA ladder sets. Samples of 100, 500, and 1000 bp were separated from a 100 bp DNA ladder (TKR-3407A, Takara Bio Inc., Otsu, Japan), and 5000 and 10,000 bp samples were obtained from a 2500 bp DNA ladder (TKR-3413A, Takara Bio Inc.) using an E-Gel electrophoresis system (G6500ST, Thermo Fisher Scientific, Waltham, MA, USA). A 48.5 kbp DNA specimen was employed as the λ-DNA (3010, TAKARA Bio Inc.). The concentration of each sample was determined by fluorometry (Qubit 2.0 System, Thermo Fisher Scientific). All DNA was purified by repeated centrifugation at 14,000× *g* for 5 min at room temperature using centrifugal filters (Amicon Ultra: ACK5003GS, Millipore, Burlington, MA, USA). The concentration of each DNA sample was adjusted to the desired value by dilution with ultra-pure water. The fluorescent dye SYBR Gold (S-11494, Thermo Fisher Scientific) was employed to visualize DNA at the final concentration of 1 µM in all cases.

### 2.3. Measurement Setup

An impedance analyzer (1260, Solartron Analytical, Hampshire, UK) fitted with a pre-amplifier (1294, Solartron Analytical) was used to carry out the electrical impedance measurements in parallel with real-time observations of the DNA with a fluorescent microscope (BX-51, Olympus, Tokyo, Japan), in conjunction with an electron multiplying charge coupled device (EM-CCD, iXon Ultra 897, Andor Technology, Belfast, UK), to record visual images. The excitation light source employed during fluorescence observations did not affect the impedance measurements, and so impedance measurements were conducted throughout the observations. The microscope stage was modified with an embedded aluminum plate for the purpose of shielding to reduce electrical noise during the impedance measurements. Data were recorded and analyzed with the Smart and Zview (Solartron Analytical, Cambridge, UK) software packages. During impedance measurements, the sampling rate was varied according to the measurement frequency during sweeping and at least 100 measurements were performed with ten different devices to evaluate the reproducibility of the measurement system. 

During each trial, the sample solution was injected into the inlet port and was then supplied to the measurement area within the electrode gap, either by capillary action or by the application of a vacuum (Hakko 349, Hakko Co., Tokyo, Japan) at the outlet port to allow for step-and-repeat single-molecule measurements. 

### 2.4. Device Fabrication

**Figure 2:**
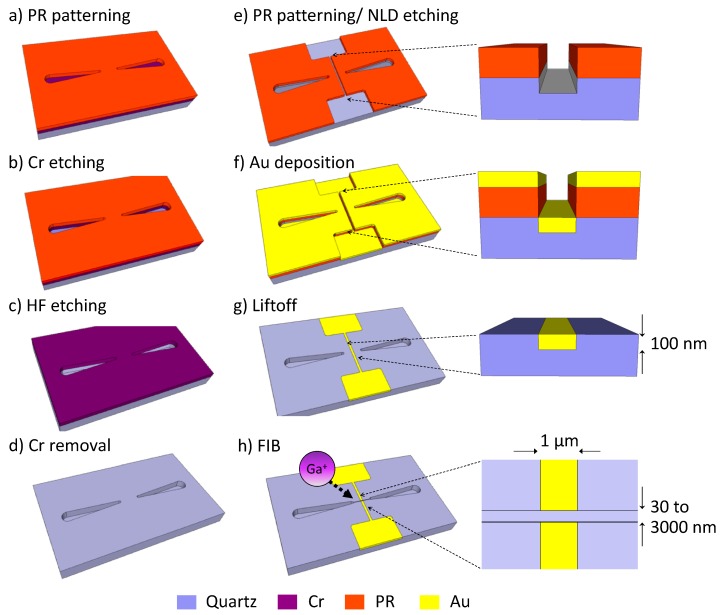
Fabrication of the measurement chip. (**a**) Photoresist (PR) patterning in preparation for the microchannel. (**b**) Etching of the Cr layer to create an etching mask to form the microchannel. (**c**) Hydrofluoric acid (HF) etching of quartz using the Cr layer as an etching mask. (**d**) Stripping of the Cr layer. (**e**) Patterning of the second photoresist followed by dry etching to create trenches for the electrode lines. (**f**) Deposition of the measurement electrode Ti/Au layers. (**g**) Stripping the photoresist layer to create the completely embedded Ti/Au electrode lines. (**h**) Simultaneous fabrication of the nanochannel and nanogap electrodes by focused ion beam (FIB) etching.

The nanofluidic measurement device was fabricated using previously published methods [24]. The microchannel was produced using a series of conventional photolithographic methods (Figure 2). In this process, a 100 nm thick Cr layer was deposited on a quartz substrate by electron beam evaporation (EBX-6D, Ulvac, Chigasaki, Japan). A photoresist (PR) layer (Figure 2a) was then used to pattern the Cr layer by conventional photolithography (Figure 2b), followed by wet etching of the quartz substrate using buffered hydrofluoric acid (BHF63U, Daikin Industries, Osaka, Japan) to create a 7 µm deep microchannel (Figure 2c,d). The second step was the fabrication of the measurement electrodes. Here, magnetic neutral loop discharge (NLD) plasma etching (NLD-570, Ulvac) was used to fabricate the trench (100 nm deep, 1 μm wide) in which the electrode was to be embedded (Figure 2e). A flow of a CF4/C3F8 gas mixture (10/10 sccm) was maintained at a total flow rate of 20 sccm under an etching pressure of 0.4 Pa and with radio frequency (RF) power of 1200 W (13.56 MHz) during the etching process. RF power (300 W, 13.56 MHz) was applied to the sample stage to generate an RF self-bias voltage. A Ti/Au (1 nm/100 nm) layer was then deposited over the substrate by electron beam sputtering (EIS 230W, Elionix, Tokyo, Japan). This process completely filled the etched trench with the Ti/Au electrode material (Figure 2f). The photoresist was removed using acetone and isopropanol to lift off the Ti/Au layer over top of the photoresist, leaving only the measurement electrode line in the trench (Figure 2g). The third step was fabrication of the nanochannel and the nanogap measurement electrodes. The nanochannel was formed by focused ion beam (FIB) etching (FB2200, Hitachi, Tokyo, Japan) (Figure 2h). A conductive film (Espacer 300Z, Showa Denko, Tokyo, Japan) was spin-coated on the substrate at 1000 rpm for 5 s followed by heating at 120 °C for 2 min to prevent charge-up during the FIB processing. FIB etching was then carried out to form the nanochannel at an accelerating voltage of 40 kV. During this process, the Ti/Au electrode strip was cut to form a pair of nanogap electrodes, generating a gap exactly equal to the nanochannel width. The cross-sectional profiles of the nanochannel and nanogap electrodes could be controlled to form a vertical to V-shaped groove by positioning the focal point of the beam onto the substrate. Finally, the substrate was sealed with a 100 μm thick layer of ultrapure PDMS (SIM 260, Shin-Etsu Silicone Inc., Tokyo, Japan) that had contact holes positioned at the microchannel ends. The PDMS was mixed with a polymerization agent in a 10:1 mass ratio. After degassing was complete, the PDMS was spin-coated at 1000 rpm for 30 s and heated at 110 °C for 120 min on a hotplate. The 100 μm thick PDMS sheet allowed the solution flow to be observed simultaneous with the measurements. The use of ultrapure PDMS in these experiments was important, because other grades of PDMS contain small amounts of silicone oil, monomers, and other contaminants that can diffuse into the nanochannel and clog the channel or induce hydrophobicity of the inner surfaces. Thus, the ultrapure silicone elastomer allowed stable flow in the nanochannel.

## 3. Results

### 3.1. Conformation Dependent Electrical Impedance Responses

The DNA molecule carries negative charges along its phosphate backbone, so in aqueous solution it can be considered as a negatively-charged polyelectrolyte. Therefore, the motion and conformation of DNA can be controlled by electrostatic forces during electrophoresis or dielectrophoresis in conjunction with electrical impedance measurements, assuming that the applied electric field is sufficiently strong. The electric field strength normally required for this purpose is on the order of several kV/m or higher, especially during dielectrophoresis, which typically requires greater than several hundred kV/m. Thus, the application of a high electric field strength is essential. 

**Figure 3:**
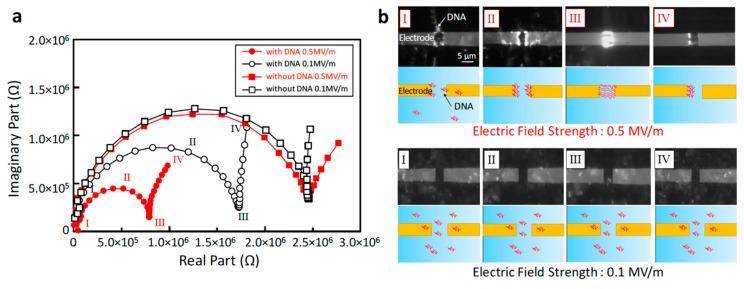
Typical electrical impedance responses and the corresponding motion and conformation of DNA. (**a**) Electrical impedance data for 10 kbp DNA (119 ng/mL) at the 3 µm electrode gap in the form of Nyquist plots. (**b**) Images of the motion and conformation of DNA from Domains I to IV in the Nyquist plots. The upper images were obtained by fluorescence microscopy and the lower images show schematic illustrations.

We initially made a qualitative investigation of the relationship between the strength of the electric field and the motion and conformation of DNA in the frequency domain by comparing fluorescent microscopy images with electrical impedance responses, as shown in Figure 3. In these trials, the DNA was visualized by labelling with a 1 µM solution of the fluorescent dye SYBR Gold (S11494, Thermo Fisher Scientific) together with a 119 ng/mL aqueous solution of DNA in pure water. In this experiment, the DNA was observed employing the glass cover plate instead of the PDMS sheet to obtain better images. In addition, the nanometer-sized gap was below the limit of optical resolution; consequently, DNA in the gap could not be observed when employing this opening. For this reason, a wider 3 µm gap was employed during visualization demonstration experiments. It should be noted that the wavelength of light (488 nm) applied to excite the fluorescent dye did not affect the impedance signal, and thus did not provide external noise during measurements. 

Figure 3a shows a typical Nyquist plot obtained from electrical impedance spectroscopy of 10 kbp DNA with a contour length of 3.4 µm approximately equal to the electrode gap of 3 µm. The impedance of a solution containing only 1 µM SYBR Gold (denoted as “without DNA”) exhibits almost the same responses at applied electric field strengths of 0.1 and 0.5 MV/m, whereas the DNA solution (denoted as “with DNA”) shows different responses. We subsequently compared the electrical impedance values obtained at electric field strengths of 0.1 and 0.5 MV/m with the visual observations of the behavior of the DNA. The numbers from I to IV in the Nyquist plot correspond to the images of the motion and conformation of the DNA shown in Figure 3b. Because the glass cover plate was floating over the electrode (Figure 1d), DNA in the vicinity of the electrode gap was attracted to or repelled by the electrode via electrostatic force during impedance measurements. Hence, the number of DNA molecules in the measurement area was not constant, resulting in measurements that were less quantitatively precise. Nonetheless, the results are sufficiently clear to allow us to distinguish the effect of the high intensity electric field by examining the motion and conformation of the DNA. 

The upper and lower parts of Figure 3b show fluorescent and schematic images of the behavior of DNA at electric field strengths of 0.1 and 0.5 MV/m, respectively. In the case of a field strength of 0.1 MV/m or less, no specific motion and conformational changes were observed. The DNA was in a random coiled conformation and underwent oscillatory fluctuations due to Brownian motion during the measurements, since no electrostatic force was exerted on the molecules. In contrast, the DNA was attracted to the electrode edges and stretched to a greater or lesser extent depending on the applied frequency in the case of a field strength of 0.5 MV/m or more. This phenomenon has been previously noted during the dielectrophoresis of DNA [25,26]. The same behavior was observed in our experiments with any of the DNA lengths we investigated, and so we believe that this behavior will be exhibited by any length of DNA.

The Nyquist plots obtained at 0.1 and 0.5 MV/m exhibit similar patterns, although the magnitude, the transition frequency at point III, and the gradient in the low frequency region IV are different. These results imply that both the resistance and capacitance components of the impedance changed simultaneously as a result of variations in the motion and conformation of the DNA. The behavior in Domain I (1–10 MHz) shows that the DNA is less attracted to the electrode edges, and has a loosely stretched conformation. In Domain II (100 kHz–1 MHz) the DNA is stretched straight out at the electrode edges, while in Domain III (10–100 kHz) the DNA is fully stretched and bridges the electrode edges. The contour length of the DNA molecules was 3.4 µm in the case of the 10 kbp sample, and so the DNA could conceivably bridge the 3 µm gap if fully stretched. In this frequency regime, the dielectrophoresis effect was strong; therefore, DNA was attracted to the electrode edges, resulting in bright white bundles of DNA bridging the electrodes. We also found that the impedance response trend underwent a drastic change in Domain III. In this region, the impedance response in the Nyquist plot changed from semicircular to nearly linear before and after transition frequency III at several tens of kHz, depending on the DNA length. After this transition frequency, the behavior in Domain IV (1–10 kHz) shows that the DNA is no longer attracted to both edges at the same time, but rather to only one electrode based on the frequency of the applied electric field. This observation indicates that the major force effect acting on the DNA may have changed from dielectrophoresis to electrophoresis. Therefore, the transition frequency represents the point at which the dominant electrostatic force transitions from dielectrophoresis to electrophoresis. Furthermore, these results suggest that we can develop an analytical model to express simultaneous dielectrophoretic and electrophoretic effects.

After withdrawing the electric field, all of the DNA was released to the solution and no molecules were observed to adhere to the electrode edges, likely due to the low level of applied voltage. Because the 3 µm electrode gap requires only 1.5 V to generate a field strength of 0.5 MV/m, any electrode reactions would be effectively suppressed; therefore, the chemisorption of DNA to the electrode would also be greatly reduced. This non-adherence characteristic would be highly advantageous with regard to quantitative analyses and also prolonged continuous measurements in future practical applications. 

In summary, visualization studies clearly revealed that an electric field strength of 0.5 MV/m or more induced changes in the motion and conformation of DNA depending on the frequency, and these variations were reflected in the impedance. 

### 3.2. Analytical Model to Evaluate the Impedance Response of DNA

**Figure 4:**
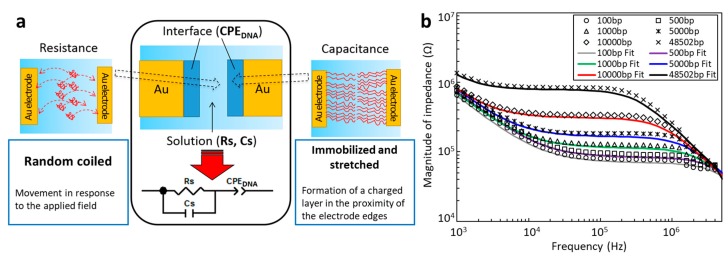
Equivalent circuit model used to quantitatively evaluate the impedance responses. (**a**) An electric circuit model for the impedance of DNA solutions in nanogap electrodes. (**b**) Fitting of the impedance responses of solutions of DNA ranging from 100 bp to 48.5 kbp in length.

To allow for quantitative evaluations, we constructed an analytical model that incorporates the impedance responses necessary to cover all of the frequency regions in the range of several kHz to 10 MHz. The measured electrical impedance values were then evaluated by extracting the constituent electrical elements using the electric circuit model shown in Figure 4a. The impedance was modeled as an electric circuit network consisting of the two major elements, resistance and capacitance, based on the visual observations of DNA behavior shown in Figure 3. In the case of the resistance element, the DNA was in a random coiled conformation and moved between electrodes according to the applied electric field in the low frequency region (below the transition frequency of several tens of kHz), as shown in Figure 3. Here, the DNA functioned as a negatively-charged carrier to increase the electric current. Therefore, the solution resistance, R_s_, is expected to be inversely proportional to both the concentration and the length of the DNA. Conversely, in the high-frequency region above the transition frequency, DNA was immobilized and stretched at the electrode edges, and thus changed the condition of the electric double layer in the proximity of the electrode edges. The equivalent circuit is, therefore, considered to involve the electric double layer capacitance based on the DNA at both electrode edges. An electric double layer capacitance system generally exhibits highly complex behavior compared with ideal capacitance because of the effects of surface roughness, chemical inhomogeneity, and highly complex ion adsorption behavior at the electrode surfaces. The immobilization of DNA should enhance these complex non-linear behaviors to produce a frequency-dependent distributed constant circuit. To satisfy this condition, a constant phase element (CPE_DNA_) was used to model the capacitance. Finally, the total impedance was modeled as the capacitance at the electrode edges (CPE_DNA_) in series with a parallel combination of the solution resistance (R_s_) and the capacitance created in the other part of the solution (C_s_) within the electrode gap. 

Figure 4b shows the results of fitting the impedance responses of DNA with lengths of 100, 500, 1 k, 5 k, 10 k, and 48.5 kbp at a concentration of 119 ng/mL. The impedance responses were obtained with a 500 nm electrode gap at an electric field strength of 0.5 MV/m with an applied voltage of 0.4 V. The components of the equivalent circuit, R_s_, C_s_, and CPE_DNA_, were extracted by a complex nonlinear least squares method using a commercially-available software package (ZView, Solartron Analytical). The reconstructed equivalent circuit values obtained with the appropriate R_s_, C_s_, and CPE_DNA_ are plotted as solid lines in Figure 4b. All of these plots are in good agreement with the measured impedance responses obtained with varying lengths of DNA. Therefore, this model should be universally applicable to the evaluation of the impedance responses of DNA solutions, and is therefore used for quantitative evaluation purposes hereafter. 

### 3.3. Single Molecular Detection of DNA

**Figure 5:**
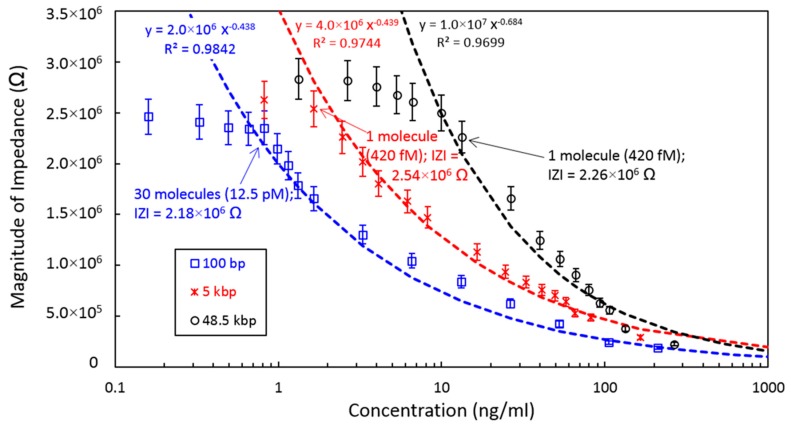
Electrical impedance values as functions of DNA concentration, based on measurements of 100 bp, 5 kbp, and 48.5 kbp DNA in a 800 nm electrode gap with an applied electric field strength of 0.5 MV/m.

Next, we attempted to achieve a level of sensitivity that would allow the single-molecule detection of DNA. For this purpose, we investigated DNA at various concentrations using a 800 nm electrode gap with the PDMS cover to confine the DNA within the nanochannel to allow for quantitative electrical impedance measurements. Figure 5 shows the magnitudes of the impedance at various concentrations from 0.3 to 1063.5 ng/mL for three lengths (100 bp, 5 kbp, and 48.5 kbp) of DNA with an applied electric field strength of 0.5 MV/m at 10 kHz. These results show that we successfully realized single-molecule detection in the case of the 5 kbp and 48.5 kbp specimens. In contrast, the detection limit was 30 molecules of DNA in the case of the 100 bp sample. All three impedance responses show that the impedance increases with decreasing concentration and tends to plateau in the approximate range of 2.2 to 2.5 MΩ. In addition, at higher concentrations, the impedance responses all converge at a value of several 10^5^ Ω. This result indicates that the detection limit at higher concentrations would be in the range of µg/mL for any length of DNA.

### 3.4. Statistical Analysis of Single-Molecule Measurements of DNA

**Figure 6:**
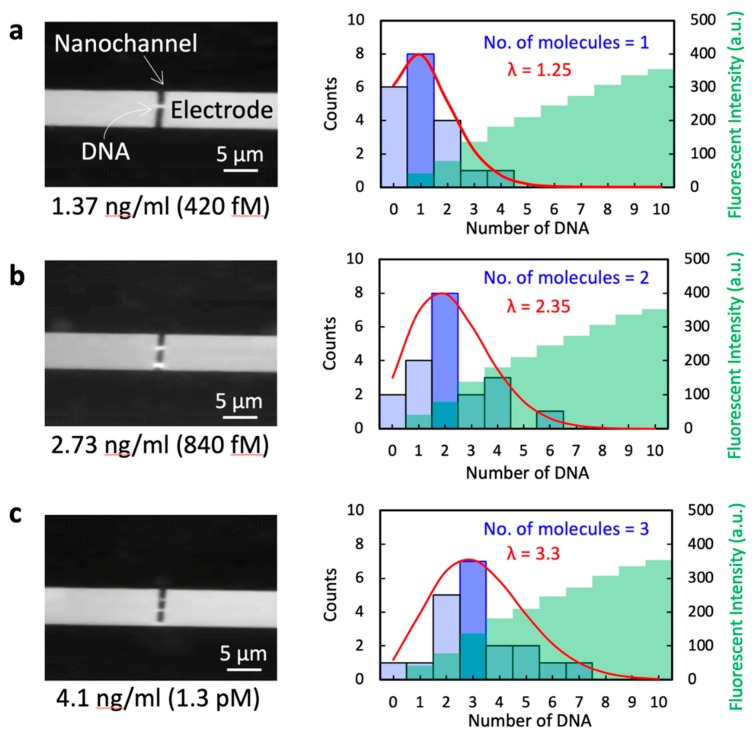
Fluorescent images of DNA in the electrode gap. These images were obtained with 5 kbp DNA in an 800 nm electrode gap at 0.5 MV/m field strength during electrical impedance measurements. The left-hand column presents fluorescence images of (**a**) single, (**b**) double, and (**c**) triple DNA molecules. The right-hand column shows the distribution histogram obtained from 20 replicate visualizations fitted to a Poisson distribution. Here, λ is the expected value of the Poisson distribution parameter. The green plots represent the corresponding histogram of the measured fluorescent intensity of each length of DNA.

In order to further investigate the single-molecule measurement of DNA, we attempted to visualize the behavior of DNA during the impedance measurement. The DNA was observed using fluorescence in conjunction with the PDMS-covered device, even though it did not allow the acquisition of DNA images as precise as those obtained with the glass cover. No DNA was present outside the electrode gap in these trials, so that only the DNA in the electrode gap was attracted to the electrode edges during the impedance measurements. The images in the left-side column in Figure 6 show the fluorescent microscopy of single, double, and triple 5 kbp DNA molecules at concentrations 420 fM, 840 fM, and 1.3 pM, respectively. These images correspond to the red plot (5 kbp DNA) in Figure 5. The plots in the right-side column show the distribution histograms obtained from 20 visualizations, fitted to Poisson distributions. These plots demonstrate that the number of DNA molecules observed is in good agreement with a Poisson process, meaning that the quantity of DNA will reflect the molar concentration. The green plot in each case represents the measured fluorescent intensity of each length of DNA. Each length is clearly distinguishable by its intensity, and the intensity values are almost linearly proportional to the length of the DNA.

These data provide evidence for the successful single-molecule measurement of DNA by both visualization and statistical analysis.

## 4. Discussion

As can be seen from Figure 5, the detection limit for a single molecule is apparently a function of the length of the DNA. The impedance values were observed to plateau at low concentrations in the case of the 5 and 48.5 kbp specimens. This phenomenon may reflect the very low probability that DNA will be present in the measurement space when the concentration is low. If the concentration is diluted to about the level of a single DNA molecule or less, the detection of DNA will be based on random chance. Thus, the impedance value at the plateau should equal that of a buffer solution at 3 MΩ, which was confirmed by removing DNA through filtration of the test samples. In contrast, the impedance plateaued at a concentration of approximately 30 molecules of DNA in the case of the 100 bp sample. Thus, the detection limit was not associated with the single-molecule concentration in the case of 100 bp DNA. In summary, the detection limit for single DNA is evidently between 100 bp to 5 kbp at the present stage of development of this system. 

**Figure 7:**
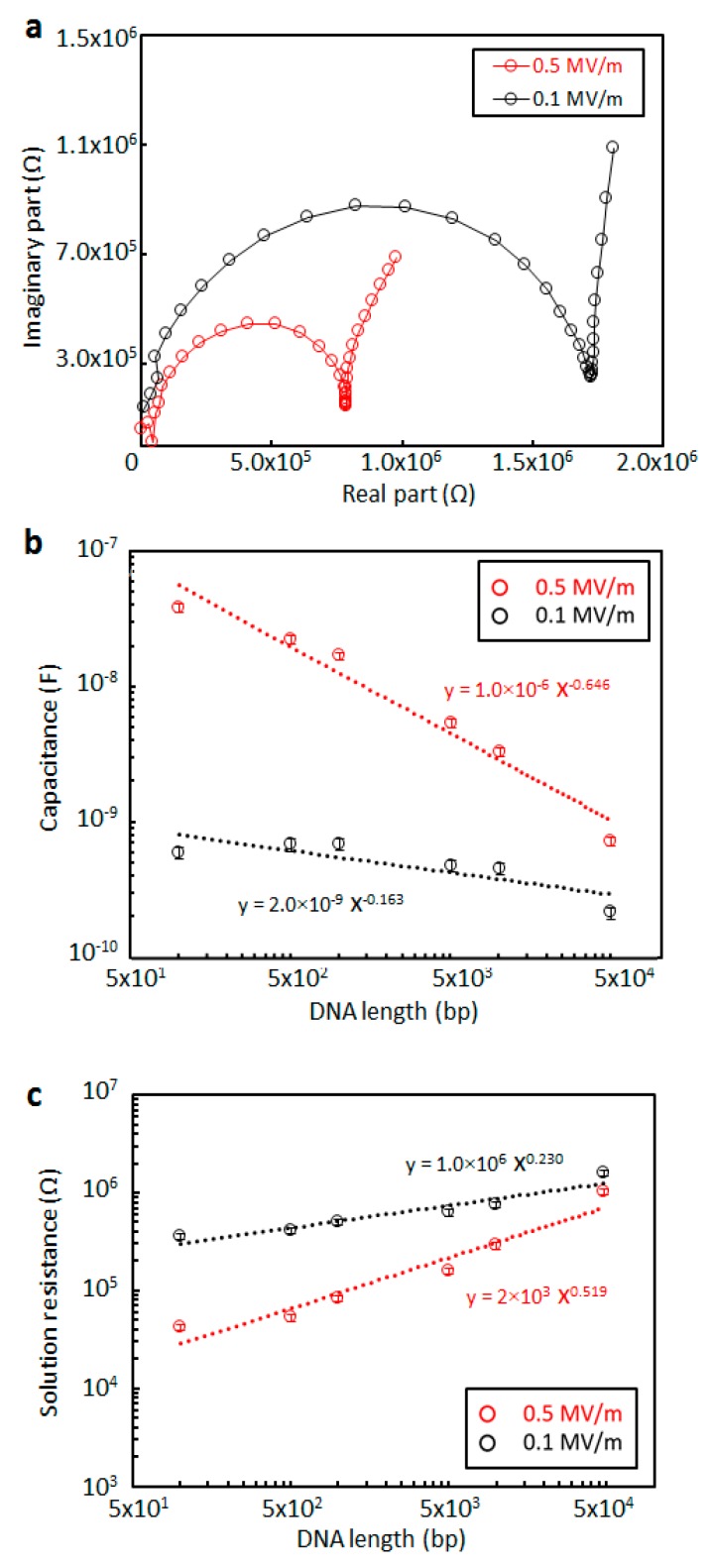
Electrical impedance values of DNA solutions as functions of length compared between 0.5 and 0.1 MV/m. (**a**) A typical Nyquist plot for 10 kbp DNA at a concentration of 119 ng/mL. (**b**) CPE_DNA_ and (**c**) R_s_ values obtained from the measured impedance of 100 bp, 500 bp, 1000 bp, 5000 bp, 10,000 bp, and 48.5 kbp DNA solutions at a concentration of 119 ng/mL using the equivalent circuit model.

Figure 7 shows the CPE_DNA_ and R_s_ values obtained from the measured impedance of DNA using the electrical circuit model shown in Figure 4a. Figure 7a presents typical Nyquist plots for the measured impedance of 10 kbp DNA at low (0.1 MV/m) and high (0.5 MV/m) electric field strengths with a 500 nm electrode gap. In this work, various lengths of DNA (100, 500, 1k, 5 k, 10 k, and 48.5 kbp) were investigated at the same concentration of 119 ng/mL, following which the CPE_DNA_ and R_s_ values were generated from the raw data using the equivalent circuit model. Figure 7b,c plot CPE_DNA_ and R_s_ at electric field strengths of 0.1 and 0.5 MV/m. The gradients at the higher electric field strength are steeper in the case of both capacitance and resistance. The CPE_DNA_ value at 0.1 MV/m is slightly decreased with increasing DNA length, whereas the CPE_DNA_ measured at 0.5 MV/m decreases more rapidly, corresponding to a sensitivity approximately 9.7 times greater than that at 0.1 MV/m. Likewise, the R_s_ value gradually increases with increasing DNA length at the electric field strength of 0.1 MV/m, but rises more rapidly at 0.5 MV/m, such that the sensitivity is about 5.8 times greater. It is clear that electrical impedance measurements at high electric field strengths (>0.5 MV/m) allow greater sensitivity in the case of both capacitance and resistance. 

The analysis time was dependent on the frequency range of the measurement, because the frequency response analyzer used in this work required a finite time span to scan over a range of frequencies. The typical measurement time in this work for a scan from 1 kHz to 10 MHz was approximately 30 s, which is greater than that required for a recently reported DC measurement system (in the range of 1–10 ms). However, it should be possible to greatly reduce this time by measuring only one or a few frequencies, if the optimum frequency required to detect the pertinent features of the sample can be identified. 

Our impedance analysis showed sensitivity on the femtomolar level, while other measurement methods using gold nanoparticles [27,28] or carbon nanotubes [29,30,31] have reported better sensitivity on the atto or zeptomolar levels. However, such systems have certain drawbacks, such as the requirement to modify a specific ligand on the detection surface, and thus cannot provide comprehensive and continuous measurements. Furthermore, these systems do not allow seamless implementation of sequential pre-processing and post-processing in a single-molecule manner. Therefore, the nanochannel-based system represents the best possible means of realizing a fully functional, single-molecule diagnostic and processing system.

Nanofluidic systems have taken a slow and steady path towards single-molecule and single-nanoparticle applications. While the technology is still being refined and is searching for its place among all the other applications currently available, the nanofluidic-based electrical impedance measurements presented herein suggest unique possibilities that could not have been imagined previously. Nanofluidic-based electrical analysis technologies can now provide more output capability than conventional electrical analysis without the need to build specialized equipment. Thus these technologies are suitable for the fabrication of mobile, wearable devices.
